# Dataset and reproducibility package for spectral-window ATR-MIR milk-protein predictions during passive warming of reconstituted skim milk

**DOI:** 10.1016/j.dib.2026.113326

**Published:** 2026-10-08

**Authors:** Kris Stefanidis, Tobias Low, Derek Long, John Billingsley

**Affiliations:** University of Southern Queensland, 487–535 West Street, Toowoomba, Queensland, 4350, Australia

**Keywords:** Chemometrics, Multivariate calibration, Kjeldahl analysis, Temperature perturbation, Technical repeatability, Open-format spectroscopy, Computational provenance

## Abstract

This data article documents the calibration, spectral, laboratory, prediction, diagnostic and computational records generated for an attenuated total reflectance mid-infrared (ATR-MIR) data collection for protein indications in reconstituted skim milk. Eight independently prepared calibration formulations were represented by one retained spectrum per formulation and by Kjeldahl protein values obtained from separate laboratory aliquots drawn from the same preparations. One additional physical preparation was measured by three technical scans at 4.10 °C and three at 20.70 °C, and a second additional preparation was measured sequentially during one passive-warming trajectory at nominal milestones of 2, 5, 8, 11, 14, 17 and 20 °C. Neither temperature-experiment preparation was Kjeldahl analysed; the 1.610% m/m value reported for the separate S2W calibration preparation is therefore identified as a formulation-associated comparator. Open-format CSV exports contain 29 spectral matrices and 52,122 long-format rows, alongside the controlled MATLAB workspace, seven one-latent-variable partial least-squares models, leave-one-formulation-out outputs, prediction tables, figure-source data, laboratory reports, software records, checksums and successful clean-run evidence. The files can be reused to inspect the spectra without proprietary software, test alternative spectral-window or calibration workflows, reproduce the deposited calculations, and examine within-trajectory temperature-associated variation and fixed-condition technical-scan dispersion within the stated experimental boundaries.

Specifications Table

**Table:** 

Subject	Engineering & Materials science
Specific subject area	Chemometric calibration and temperature-associated variation in ATR-MIR dairy-protein measurements
Type of data	Raw and processed spectra; long-format CSV matrices; calibration and prediction tables; MATLAB data and code; PLS models and diagnostics; figures; laboratory reports; metadata; checksums and run logs
Data collection	Eight independent calibration formulations each supplied one retained spectrum; a separate aliquot from each preparation was analysed by Kjeldahl. One further preparation supplied three technical scans at 4.10 °C and three at 20.70 °C. A second further preparation supplied one seven-point passive-warming trajectory (2–20 °C). ATR-MIR: ReactIR 702 L, diamond ATR, iC IR 7.0.
Data source location	University of Southern Queensland, Toowoomba, Queensland, Australia; independent Kjeldahl analysis by DTS/BVAQ Laboratories, Kensington, Melbourne, Australia
Data accessibility	Repository name: Mendeley Data Data identification number: 10.17632/mf2pgbjrbd.4 (Version 4; published 18 September 2026) Direct URL: https://doi.org/10.17632/mf2pgbjrbd.4 Access: CC BY 4.0; open access.
Related research article	K. Stefanidis, T. Low, D. Long, J. Billingsley, Diagnosing temperature-induced bias and dispersion in ATR-MIR protein measurement using spectral-window PLS models, under review in Measurement following major revision, manuscript MEAS-d-26–14100R1 [1].

## 1 Value of the Data

• Open-format calibration, passive-warming and fixed-condition spectra provide sample identifiers, wavenumbers in cm⁻¹, absorbance in absorbance units and formulation metadata, allowing inspection and reuse without MATLAB.
• Explicit preparation and replicate labels distinguish eight independent calibration formulations from one seven-observation warming trajectory and from three technical scans at each fixed condition, supporting reuse without treating model outputs or repeated scans as additional independent samples.
• Calibration matrices, measured Kjeldahl values, fixed spectral-window definitions, leave-one-formulation-out predictions and latent-variable diagnostics can be used to evaluate alternative preprocessing, spectral subsets and parsimonious chemometric workflows within the eight-formulation design.
• The temperature-experiment files support descriptive analysis of variation along one passive-warming trajectory, short-term dispersion among fixed-condition technical scans, and deviation from a clearly labelled formulation-associated protein comparator; they do not require an assumption of direct sample-specific reference analysis.
• Repository-contained MATLAB code, a one-command public runner, software metadata, run logs, source and output hashes, a canonical-file map and a file-level SHA-256 manifest permit computational auditing and adaptation of the workflow.

## 2 Background

ATR-MIR spectroscopy has been applied to milk-protein measurement because aqueous spectra contain composition-related information that can be related to reference protein values by multivariate calibration [2]. Temperature can also alter band position, intensity, baseline structure, hydrogen bonding, water-band structure, viscosity and sample–sensor contact without changing the protein measurand [3,4]. The deposited records were compiled to expose those measurement-state effects and the associated computational workflow. They comprise eight independently referenced calibration preparations, one separate fixed-condition preparation and one separate passive-warming preparation. The two temperature-experiment preparations share the nominal S2W formulation and powder batch with the calibration series but were not Kjeldahl analysed; the S2W laboratory value is therefore a formulation-associated comparator. The related Measurement article interprets the spectral-window modelling results [1], whereas this article describes the underlying data, provenance, software and reuse boundaries.

## 3 Data Description

The corrected repository was published as Mendeley Data Version 4 on 18 September 2026 at https://doi.org/10.17632/mf2pgbjrbd.4 [5]. The package uses the repository title “ATR-MIR spectral data and PLS outputs for protein measurement in reconstituted skim milk during passive warming”, is licensed CC BY 4.0, and contains 182 files in 15 top-level folders. The file-level SHA-256 manifest contains 181 data rows because the manifest does not hash itself.

The top-level file counts and functions are: 00_README (11; overview, citation, dictionary, matrix and canonical-file maps, experimental metadata, licence and software notes); 01_Calibration (14; formulation/reference table, source workspace and open-format calibration matrices); 02_Raw_Spectra (26; passive-warming and fixed-condition spectral CSVs and their manifests/readmes); 03_Processed_Spectra (1; 20 °C minus 2 °C difference spectrum); 04_PLS_Models (31; rebuilt models, coefficients, loadings and diagnostic graphics); 05_Prediction_Outputs (2; warming and fixed-condition long tables); 06_MATLAB_Code (12; ten scripts, code archive and runner guide); 07_Analysis_Outputs (15; model, cross-validation, latent-variable, prediction, metric and correction tables plus MAT output); 08_Figure_Source_Data (6); 09_Final_Figures (26); 10_Supplementary_Material (2); 11_Laboratory_Reference (2; complete reports and guide); 12_Manifest_and_Logs (11); 14_Reproduction_Run (22; clean-run and public-run evidence); and 99_Excluded_Legacy (1; scope-boundary note). README.md, CANONICAL_FILE_MAP.csv and FILE_MANIFEST_SHA256.csv provide file-by-file roles, aliases and hashes (Table 1, Table 2).

**Table 1:** Experimental units, repeated observations and effective sample sizes.

Component	Physical unit and observations	Effective n and analytical use
Calibration series	Eight independently prepared formulations (S1W–S7W, S9W); one retained spectrum per preparation; separate laboratory aliquot from the same preparation.	n = 8 formulations per model; eight leave-one-formulation-out folds, with one complete formulation withheld per fold.
Fixed-condition series	One separate S2W-nominal physical preparation measured at 4.10 and 20.70 °C; three technical scans at each condition.	n = 3 scans per condition and model, but one preparation. The 42 predictions (7 models × 2 conditions × 3 scans) are not 42 independent samples.
Passive-warming series	A second separate S2W-nominal physical preparation measured sequentially at 2, 5, 8, 11, 14, 17 and 20 °C.	n = 7 ordered observations per model within one trajectory. The 49 predictions (7 models × 7 milestones) are not independent temperature replicates.
Internal correction	Seven PhosCov warming predictions from the single trajectory; each calculation fits six points and omits one.	Seven leave-one-temperature-out calculations; experimental independence remains one physical trajectory.
Models and duplicated outputs	Seven spectral-window models applied repeatedly to the same source observations; one exact Amide ALL calibration-matrix duplicate is retained for provenance.	Models, predictions, plots, formats and aliases do not increase the number of independent preparations.

**Table 2:** Principal scientific matrices, dimensions, units and relationships.

Scientific object	Dimensions, variables and units	Canonical location and relationship
Calibration response	8 × 1; Kjeldahl protein, % m/m; formulation IDs S1W–S7W and S9W.	01_Calibration/Calibration_Formulations_and_Kjeldahl.csv; measured laboratory response for the eight calibration preparations.
Calibration spectra	Seven active 8 × p matrices, p = 1654, 20, 49, 50, 75, 282 and 244; absorbance (AU) versus descending wavenumber (cm⁻¹). One additional 8 × 282 Amide ALL matrix is an exact deposited duplicate and is not active.	01_Calibration/Calibration_Spectral_CSV/*.csv; 8 exports and 21,248 long rows. Sample IDs and Kjeldahl mapping are included.
Passive-warming spectra	Seven 7 × p matrices using the same p sequence; AU versus cm⁻¹; nominal milestones 2–20 °C; one physical trajectory.	02_Raw_Spectra/Warming_Spectral_CSV/*.csv; 7 exports and 16,618 long rows; linked to TS5_06/TS5_SMP_*_VarTemp sources.
Fixed-condition spectra	Fourteen 3 × p matrices (7 windows × 2 conditions). Full MIR has 1656 deposited points; two high-wavenumber points not present in the 1654-variable calibration grid are retained but excluded by exact value matching during model application.	02_Raw_Spectra/Fixed_Condition_Spectral_CSV/*.csv; 14 exports and 14,256 long rows. Fixed PhosCov TS0486 files are a separate acquisition sequence from the same physical preparation, not Full-MIR subsets.
Predictions	49 warming rows and 42 fixed-condition rows; model, condition/temperature, observation/scan, prediction and comparator-deviation fields; protein in % m/m and deviations in percentage points.	05_Prediction_Outputs/*.csv. Legacy Benchmark_percent denotes the 1.610% formulation-associated comparator, not a direct assay of either temperature sample.
Calibration and LV diagnostics	56 leave-one-formulation-out rows; 21 LV1–LV3 RMSECV rows; 21 LV1–LV3 X/Y variance rows; seven model-summary rows.	07_Analysis_Outputs/01_*, 02_*, 03_* and 13_*. These are model-derived outputs, not additional observations.

The 29 open-format spectral matrices contain 52,122 long-format rows: eight calibration exports (21,248 rows, including the labelled exact Amide ALL duplicate), seven passive-warming exports (16,618 rows) and 14 fixed-condition exports (14,256 rows). Spectral values and axes are finite, axes are unique and strictly descending, and the repository uses blank text fields only where text is inapplicable. In the leave-one-formulation-out table, NaN in LOFO_Prediction_SD_percent is intentional because each fold has one withheld spectrum and therefore no within-fold prediction SD. MATRIX_INVENTORY.csv gives all 30 X/Y matrix records, exact axes and experimental units; CANONICAL_FILE_MAP.csv distinguishes canonical files from derived or intentional duplicate representations.

The source-to-derived path is: the controlled workspace and calibration map in 01_Calibration feed the scripts in 06_MATLAB_Code; the scripts rebuild the seven models in 04_PLS_Models, apply them to the spectra in 02_Raw_Spectra, and generate 05_Prediction_Outputs, 07_Analysis_Outputs, 08_Figure_Source_Data and 09_Final_Figures. The complete laboratory evidence is in 11_Laboratory_Reference, and 12_Manifest_and_Logs plus 14_Reproduction_Run record source/output hashes and successful validation. The MAT files preserve native PLS_Toolbox objects; the corresponding CSV exports provide software-independent access to spectral values, coefficients, intercepts, predictions and diagnostics.

## 4 Experimental Design, Materials and Methods

### 4.1 Experimental units and preparation relationships

Three sets of liquids were prepared from the same commercially sourced skim-milk-powder batch. The calibration set comprised eight independently prepared formulations, with one retained spectrum per formulation. A separate aliquot drawn from each prepared formulation was submitted for Kjeldahl analysis; those laboratory aliquots were not pre-scanned. The fixed-condition set comprised one separately prepared liquid at the nominal S2W formulation, measured at two equilibrated conditions. The passive-warming set comprised a second separately prepared liquid at the same nominal formulation, measured sequentially as it warmed. Neither temperature-experiment liquid was Kjeldahl analysed. The 1.610% m/m S2W result therefore belongs to a separate calibration preparation and is used only as a formulation-associated comparator.

### 4.2 Materials, storage and gravimetric preparation

Bulk skim-milk powder was stored at approximately −22 °C, transferred to approximately 4 °C for 24 h, and then used to prepare liquids. Prepared liquids were refrigerated overnight at approximately 4 °C. Masses were measured with a Mettler Toledo PC440 balance, with reported readability of 0.01 g in the standard range and 0.001 g in the fine range. Glass vessels of 100, 200 and 500 mL capacity were used as appropriate. Powder was added first, followed by distilled water. Mixing used a Schott Instruments magnetic-stirring unit marked “D-55,122 Mainz”, in mixing mode at the 25% controller setting during formulation and temperature-experiment acquisition. The exact model number and a calibrated rotational speed in rpm were not recorded. Temperature-experiment liquids were uncovered and mixed continuously during acquisition.

### 4.3 Calibration formulations and laboratory analysis

The calibration preparations S1W–S7W and S9W spanned powder-to-water mass ratios of 1.00–18.06%, calculated as 100 × powder mass/water mass. Kjeldahl protein results spanned 0.34–5.07% m/m. DTS/BVAQ Laboratories reported protein using method PROT 01 02.01 (repository code PROT010201), calculated as total nitrogen × 6.38. The complete evidence bundle contains both pages of the 2018 liquid-sample report and the associated powder-batch report, which reports 33.2% m/m protein. The reports do not state sample-specific replicate counts, quality-control results or contemporaneous measurement uncertainty. A later laboratory statement gives ±2.6% relative uncertainty for the same method based on recent data; this contextual value was not assigned retrospectively to the 2018 results.

### 4.4 ATR-MIR acquisition and spectral treatment

Spectra were acquired with a Mettler Toledo ReactIR 702 L FT-MIR system, a diamond ATR element and Sentinel optical pack, using iC IR version 7.0. Each spectrum comprised 256 co-added scans at nominal 4 cm⁻¹ resolution. The stored Full-MIR calibration grid contains 1654 unique descending wavenumbers from 3993.3552 to 599.5166 cm⁻¹ at approximately 2.05314 cm⁻¹ intervals; absorbance is reported in absorbance units (AU). Sample temperature was measured during temperature-experiment scans with a Testo 108 thermometer (reported range −50 to 300 °C, accuracy ±0.5 °C and resolution 0.1 °C). Open-air background subtraction was used. No additional atmospheric correction, baseline correction, smoothing, interpolation or spectral normalisation was applied to the exported raw spectra. Calibration-spectrum temperatures were not recorded in a form suitable for retrospective assignment.

### 4.5 Fixed-condition acquisition

The fixed-condition liquid was equilibrated at 4.10 °C for 1 h. The ATR probe was cleaned with acetone, inserted into the continuously mixed liquid, and used to acquire three technical spectra. The probe was then removed and cleaned with acetone. The same liquid was allowed to warm passively to 20.70 °C, held at that temperature for 1 h, and measured by three further technical scans after reinsertion of the cleaned probe. The Full-MIR and Amide objects retain TS0506 source labels. The PhosCov objects retain legacy TS0486 labels and constitute a separate PhosCov acquisition sequence from the same physical fixed-condition preparation; they are not the same individual scans as, or exact column subsets of, the TS0506 Full-MIR scans.

### 4.6 Passive-warming acquisition

The separate passive-warming liquid was removed from refrigeration at approximately 2 °C. The probe was cleaned with acetone before insertion and remained immersed without removal or cleaning while the uncovered liquid was mixed continuously and warmed passively. One spectrum was retained when the measured sample temperature reached each nominal milestone of 2, 5, 8, 11, 14, 17 and 20 °C. Temperature was read during each scan. Warming was gradual relative to spectral acquisition frequency, but elapsed times at the milestones were not recorded. The seven spectra are therefore one ordered trajectory rather than seven independent temperature treatments.

### 4.7 Spectral windows and PLS modelling

Seven fixed spectral windows were used. Exact descending calibration axes were: Full MIR, 3993.3552–599.5166 cm⁻¹ (1654 variables); PhosCov, 1100.4825–1061.4728 cm⁻¹ (20); Amide I, 1699.9990–1601.4484 cm⁻¹ (49); Amide II, 1601.4484–1500.8446 cm⁻¹ (50), with 1601.4484 cm⁻¹ included in both Amide I and II; Amide III, 1350.9654–1199.0331 cm⁻¹ (75); Amide I_II_III, 1699.9990–1201.0863 cm⁻¹ (244); and Amide ALL, 282 variables in six discontinuous segments (3299.3943–3278.8629, 3104.3461–3085.8678, 1699.9990–1500.8446, 1350.9654–1201.0863, 1100.4825–1061.4728 and 769.9271–630.3137 cm⁻¹). The exact vectors, rather than rounded nominal endpoints, define inclusion.

SIMPLS models [6,7] used X mean centring and single-response Y autoscaling. In each leave-one-formulation-out fold, all fitted transformations and model parameters were estimated using only the seven training formulations and then applied to the omitted formulation. One latent variable was fixed a priori for all windows because only eight independent formulations were available; a common one-LV complexity limited overfitting and preserved a like-for-like window comparison. Separate one-to-three-LV sensitivity files were retained but did not replace the active models. In the all-eight-formulation diagnostic fits, LV1 captured 98.6797–99.9953% of X variance and 99.1749–99.8986% of Y variance across the seven windows. One-LV leave-one-formulation-out RMSECV ranged from 0.069934 to 0.235441 protein percentage points. Higher-LV sensitivity minima occurred for PhosCov at LV2 (0.041052) and Amide II at LV3 (0.163918), but were not promoted because selecting unequal complexity on n = 8 would increase selection instability. For a single response, Y autoscaling is multiplication by one constant; it does not balance multiple responses and, after back-transformation, leaves latent directions and predictions equivalent to centred-Y fitting within numerical precision. Predictions and error metrics were returned to % m/m and percentage-point units.

### 4.8 Descriptive metrics and sample counts

For predictions yᵢ, sample SD was s = [Σ(yᵢ − ȳ)²/(n − 1)]^1/2^, CV was 100s/ȳ (%), and range was max(yᵢ) − min(yᵢ) (protein percentage points). For the temperature-experiment outputs, c = 1.610% m/m denotes the formulation-associated comparator and dᵢ = yᵢ − c denotes signed comparator deviation. Mean signed comparator deviation was (1/n)Σdᵢ, mean absolute comparator deviation was (1/n)Σ|dᵢ|, and root-mean-square comparator deviation was [(1/n)Σdᵢ²]^1/2^, all in percentage points. Legacy files retain the labels Bias, MAE, RMSE or Benchmark for these quantities; those labels do not indicate sample-specific reference analysis. For calibration, RMSECV = [(1/8)Σ(ŷ₋ᵢ − yᵢ)²]^1/2^ used the measured Kjeldahl value of each omitted formulation. Effective n was 8 independent formulations per model for calibration, 7 sequential observations per model for warming, and 3 technical scans per condition and model for fixed-condition dispersion. No inferential test treated models, temperatures or scans as independent preparations.

### 4.9 Illustrative internal residual–temperature calculation

The PhosCov series supplied an internal leave-one-temperature-out calculation. For omitted observation j, deviations dᵢ = yᵢ − c from the remaining six warming observations were fitted as dᵢ = a₋ⱼ + b₋ⱼTᵢ. The corrected omitted prediction was yⱼ,corr = yⱼ − (a₋ⱼ + b₋ⱼTⱼ). This was repeated for all seven observations. A quadratic relation was retained as a sensitivity output. The calculation uses the same seven-point trajectory and formulation-associated comparator for fitting and checking; it is an illustrative internal data product, not an externally validated or transferable compensation model.

### 4.10 Computational reproduction and integrity records

The reference environment is MATLAB R2024b with Eigenvector Research PLS_Toolbox 9.5.1. From 06_MATLAB_Code, a clean session clears the workspace and then runs MEASUREMENT_RUN_REPOSITORY. The self-locating runner verifies controlled source hashes, rebuilds the seven models, regenerates tables, predictions, spectra, diagnostics and figures, and writes results to a new timestamped folder without modifying deposited files. A clean public run on 17 September 2026 returned PASS, generated 71 scientific artifacts and produced a 71-row SHA-256 artifact manifest with no missing or mismatched files. The open-format exporter independently returned PASS for all 29 matrices and 52,122 long-format rows; its 38 output hashes and three controlled-source hashes were rechecked. The success record, complete log and artifact manifest are deposited in 14_Reproduction_Run/Public_Run_20,260,917_121,551, and open-format validation records are in 12_Manifest_and_Logs/Open_Format_Spectra_Export.

## Limitations

The data describe one commercially sourced skim-milk-powder batch, eight calibration preparations, one fixed-condition preparation, one separate passive-warming preparation and one ATR-MIR instrument. The seven warming observations are sequential measurements from one liquid; temperature is confounded with elapsed time because milestone times were not recorded. The three scans at each fixed condition are technical scans of the same liquid, not preparation replicates. Neither temperature-experiment liquid was Kjeldahl analysed, so 1.610% m/m is a formulation-associated comparator rather than a sample-specific reference. Calibration-spectrum temperatures were not recoverable. The 2018 reports do not provide sample-specific analytical replication, quality-control or measurement-uncertainty records; a later laboratory statement of ±2.6% relative uncertainty for the same method was based on recent data and was not assigned retrospectively. The fixed PhosCov spectra retain legacy TS0486 labels and form a separate window acquisition sequence from the TS0506 Full-MIR/Amide scans. The package does not sample between-batch, natural-matrix, between-day, between-instrument or industrial-process variability.

## Ethics Statement

The authors have read and followed the ethical requirements for publication in Data in Brief. The work did not involve human subjects, live-animal experiments or data collected from social media platforms.

## CRediT Author Statement

**Kris Stefanidis:** Conceptualization; Methodology; Investigation; Data curation; Formal analysis; Software; Validation; Visualization; Project administration; Writing – original draft; Writing – review & editing; **Tobias Low:** Supervision; Methodology; Validation; Writing – review & editing; **Derek Long:** Supervision; Methodology; Validation; Writing – review & editing; **John Billingsley:** Supervision; Writing – review & editing.

## Author Approval

All authors have approved the revised manuscript and its resubmission to Data in Brief.

## Data Availability

Mendeley DataATR-MIR spectral data and PLS outputs for protein measurement in reconstituted skim milk during passive warming (Original data).

## Appendix Supplementary materials

Image, application 1
