# Supplementary material for: Dataset and reproducibility package for spectral-window ATR-MIR milk-protein predictions during passive warming of reconstituted skim milk
Source: Data Brief. 2026 Oct 8;69:113326. doi: 10.1016/j.dib.2026.113326 (PMC13695134; doi:10.1016/j.dib.2026.113326)
Supplement: Supplementary file 1 [file mmc1.docx]

**Supplementary material**

**Dataset and reproducibility package for spectral-window ATR-MIR milk-protein predictions during passive warming of reconstituted skim milk**

Kris Stefanidis, Tobias Low, Derek Long and John Billingsley

Data in Brief manuscript DIB-D-26-02195

Repository title: ATR-MIR spectral data and PLS outputs for protein measurement in reconstituted skim milk during passive warming

Mendeley Data, Version 4; https://doi.org/10.17632/mf2pgbjrbd.4

Supplementary Tables S0A-S0B, S1-S6, S7A-S7C and S8, together with Supplementary Figures S1-S4, document the calibration formulations, passive-warming observations, fixed-condition technical scans, model diagnostics and internal sensitivity calculations described in the Data in Brief article. Values are generated from the corrected repository and the successful public reproduction run in MATLAB R2024b with PLS_Toolbox 9.5.1.

**Supplementary Table S0A. Dataset structure, experimental units and effective sample sizes.**

| **Data block** | **Physical material** | **Recorded observations** | **Effective n and interpretation** |
| --- | --- | --- | --- |
| Calibration | Eight independently prepared formulations S1W-S7W and S9W; laboratory aliquots came from the same respective preparations. | One retained spectrum per formulation and eight Kjeldahl values. | n = 8 independent formulations; leave-one-formulation-out validation used eight folds. |
| Fixed condition | One additional S2W-nominal liquid preparation measured first at 4.10 °C and later at 20.70 °C. | Three technical scans at each condition; the probe was cleaned and reinserted between the refrigerated and ambient stages. | n = 1 preparation and n = 3 scan indications per condition; scans quantify technical dispersion, not biological replication. |
| Passive warming | A second additional S2W-nominal liquid preparation. | Seven ordered observations at nominal 2, 5, 8, 11, 14, 17 and 20 °C during one continuous warming trajectory. | n = 1 preparation and n = 1 trajectory; temperature is confounded with elapsed time and milestone times were not recorded. |
| Derived outputs | No additional physical samples. | Seven model predictions per observation, diagnostics, plots, alternate formats and internal residual-temperature calculations. | Derived and duplicated representations are not additional independent observations; the correction is illustrative and internal. |

Neither temperature-experiment preparation was Kjeldahl analysed. The 1.6100% m/m value is the Kjeldahl result for the separate S2W calibration preparation and is used only as a formulation-associated comparator. Sample SD uses n-1; CV is 100 times SD divided by the mean; range is maximum minus minimum; signed comparator deviation is prediction minus comparator; mean absolute comparator deviation averages the absolute deviations; and root-mean-square comparator deviation is the square root of the mean squared deviations. These quantities distinguish calibration error, fixed-condition technical-scan dispersion and within-trajectory variation. They do not constitute a complete measurement-uncertainty budget, external validation or transferable temperature compensation.

Supplementary Table S1. Individual indicated protein concentrations during the low-solids passive-warming sequence.

| **Temperature (°C)** | **Full MIR** | **PhosCov** | **Amide I** | **Amide II** | **Amide III** | **Amide ALL** | **Amide I_II_III** |
| --- | --- | --- | --- | --- | --- | --- | --- |
| 2.0 | 1.5665 | 1.5535 | 1.6621 | 1.7058 | 1.5530 | 1.5638 | 1.6305 |
| 5.0 | 1.5957 | 1.5698 | 1.5763 | 1.6037 | 1.5800 | 1.6001 | 1.5857 |
| 8.0 | 1.5703 | 1.5518 | 1.4114 | 1.5289 | 1.5125 | 1.5423 | 1.5008 |
| 11.0 | 1.6235 | 1.5719 | 1.5802 | 1.6689 | 1.6292 | 1.6355 | 1.6401 |
| 14.0 | 1.5704 | 1.5604 | 1.5255 | 1.6169 | 1.5816 | 1.5351 | 1.5834 |
| 17.0 | 1.6145 | 1.5669 | 1.6487 | 1.7179 | 1.6231 | 1.6140 | 1.6528 |
| 20.0 | 1.4941 | 1.5076 | 1.4823 | 1.4686 | 1.4307 | 1.4579 | 1.4555 |

Note. Values are indicated protein concentrations (% m/m) from the retained one-latent-variable PLS models. The seven rows are ordered observations from one physical passive-warming preparation and are not independent temperature replicates.

Supplementary Table S2. Individual indicated protein concentrations from fixed-condition technical scans.

| **Condition** | **Temperature (°C)** | **Scan** | **Full MIR** | **PhosCov** | **Amide I** | **Amide II** | **Amide III** | **Amide ALL** | **Amide I_II_III** |
| --- | --- | --- | --- | --- | --- | --- | --- | --- | --- |
| Ambient | 20.7 | 1 | 1.6417 | 1.5921 | 2.6252 | 2.0088 | 1.7530 | 1.8664 | 2.0131 |
| Ambient | 20.7 | 2 | 1.6094 | 1.5919 | 2.6672 | 1.9961 | 1.7634 | 1.8117 | 2.0303 |
| Ambient | 20.7 | 3 | 1.6478 | 1.6216 | 2.6681 | 2.0149 | 1.8154 | 1.8569 | 2.0648 |
| Refrigerated | 4.1 | 1 | 1.6736 | 1.5979 | 2.5389 | 2.0984 | 1.7001 | 1.9075 | 2.0015 |
| Refrigerated | 4.1 | 2 | 1.6477 | 1.6150 | 2.6774 | 2.0438 | 1.6869 | 1.8617 | 2.0017 |
| Refrigerated | 4.1 | 3 | 1.6422 | 1.6099 | 2.6811 | 2.0683 | 1.6650 | 1.8718 | 1.9942 |

Note. Each condition contains three technical scans of the same physical fixed-condition liquid, not three independent preparations. Full MIR and Amide results use TS0506-labelled scans; PhosCov uses the separate TS0486-labelled acquisition sequence from the same preparation. Rows are aligned by condition and scan index for display and do not denote identical individual spectra across those acquisition sequences.

Supplementary Table S3. Descriptive statistics for the low-solids passive-warming sequence.

| **Model** | **n** | **Mean indicated protein (% m/m)** | **SD (percentage points)** | **CV (%)** | **Minimum (% m/m)** | **Maximum (% m/m)** | **Range (percentage points)** | **Mean signed comparator deviation (percentage points)** | **Mean absolute comparator deviation (percentage points)** | **Root-mean-square comparator deviation (percentage points)** |
| --- | --- | --- | --- | --- | --- | --- | --- | --- | --- | --- |
| Full MIR | 7 | 1.5764 | 0.0428 | 2.716 | 1.4941 | 1.6235 | 0.1295 | −0.0336 | 0.0387 | 0.0520 |
| PhosCov | 7 | 1.5546 | 0.0221 | 1.422 | 1.5076 | 1.5719 | 0.0643 | −0.0554 | 0.0554 | 0.0591 |
| Amide I | 7 | 1.5552 | 0.0896 | 5.758 | 1.4114 | 1.6621 | 0.2507 | −0.0548 | 0.0807 | 0.0994 |
| Amide II | 7 | 1.6158 | 0.0919 | 5.689 | 1.4686 | 1.7179 | 0.2493 | +0.0058 | 0.0712 | 0.0853 |
| Amide III | 7 | 1.5586 | 0.0691 | 4.434 | 1.4307 | 1.6292 | 0.1985 | −0.0514 | 0.0607 | 0.0821 |
| Amide ALL | 7 | 1.5641 | 0.0598 | 3.826 | 1.4579 | 1.6355 | 0.1777 | −0.0459 | 0.0543 | 0.0719 |
| Amide I_II_III | 7 | 1.5784 | 0.0744 | 4.716 | 1.4555 | 1.6528 | 0.1973 | −0.0316 | 0.0583 | 0.0758 |

Note. n = 7 ordered observations from one physical preparation and one passive-warming trajectory; these are not independent temperature replicates.

Supplementary Table S4. Descriptive statistics for low-solids fixed ambient technical scans.

| **Model** | **n** | **Mean indicated protein (% m/m)** | **SD (percentage points)** | **CV (%)** | **Minimum (% m/m)** | **Maximum (% m/m)** | **Range (percentage points)** | **Mean signed comparator deviation (percentage points)** | **Mean absolute comparator deviation (percentage points)** | **Root-mean-square comparator deviation (percentage points)** |
| --- | --- | --- | --- | --- | --- | --- | --- | --- | --- | --- |
| Full MIR | 3 | 1.6330 | 0.0207 | 1.265 | 1.6094 | 1.6478 | 0.0384 | +0.0230 | 0.0234 | 0.0285 |
| PhosCov | 3 | 1.6019 | 0.0171 | 1.065 | 1.5919 | 1.6216 | 0.0296 | −0.0081 | 0.0158 | 0.0161 |
| Amide I | 3 | 2.6535 | 0.0245 | 0.923 | 2.6252 | 2.6681 | 0.0429 | +1.0435 | 1.0435 | 1.0437 |
| Amide II | 3 | 2.0066 | 0.0096 | 0.477 | 1.9961 | 2.0149 | 0.0188 | +0.3966 | 0.3966 | 0.3967 |
| Amide III | 3 | 1.7773 | 0.0334 | 1.881 | 1.7530 | 1.8154 | 0.0624 | +0.1673 | 0.1673 | 0.1695 |
| Amide ALL | 3 | 1.8450 | 0.0292 | 1.584 | 1.8117 | 1.8664 | 0.0547 | +0.2350 | 0.2350 | 0.2362 |
| Amide I_II_III | 3 | 2.0361 | 0.0263 | 1.293 | 2.0131 | 2.0648 | 0.0517 | +0.4261 | 0.4261 | 0.4266 |

Note. n = 3 technical scans from one physical preparation at 20.70 °C, not three independent samples.

Supplementary Table S5. Descriptive statistics for low-solids fixed refrigerated technical scans.

| **Model** | **n** | **Mean indicated protein (% m/m)** | **SD (percentage points)** | **CV (%)** | **Minimum (% m/m)** | **Maximum (% m/m)** | **Range (percentage points)** | **Mean signed comparator deviation (percentage points)** | **Mean absolute comparator deviation (percentage points)** | **Root-mean-square comparator deviation (percentage points)** |
| --- | --- | --- | --- | --- | --- | --- | --- | --- | --- | --- |
| Full MIR | 3 | 1.6545 | 0.0167 | 1.012 | 1.6422 | 1.6736 | 0.0313 | +0.0445 | 0.0445 | 0.0465 |
| PhosCov | 3 | 1.6076 | 0.0088 | 0.546 | 1.5979 | 1.6150 | 0.0171 | −0.0024 | 0.0057 | 0.0076 |
| Amide I | 3 | 2.6325 | 0.0811 | 3.079 | 2.5389 | 2.6811 | 0.1422 | +1.0225 | 1.0225 | 1.0246 |
| Amide II | 3 | 2.0702 | 0.0273 | 1.321 | 2.0438 | 2.0984 | 0.0546 | +0.4602 | 0.4602 | 0.4607 |
| Amide III | 3 | 1.6840 | 0.0177 | 1.052 | 1.6650 | 1.7001 | 0.0351 | +0.0740 | 0.0740 | 0.0754 |
| Amide ALL | 3 | 1.8803 | 0.0240 | 1.279 | 1.8617 | 1.9075 | 0.0458 | +0.2703 | 0.2703 | 0.2710 |
| Amide I_II_III | 3 | 1.9991 | 0.0042 | 0.212 | 1.9942 | 2.0017 | 0.0074 | +0.3891 | 0.3891 | 0.3891 |

Note. n = 3 technical scans from one physical preparation at 4.10 °C, not three independent samples.

Supplementary Table S6. Sensitivity of warming-sequence mean signed deviation and root-mean-square deviation to the selected comparator value.

| **Model** | **Separate S2W calibration-preparation comparator (1.6100% m/m): mean signed deviation** | **Separate S2W calibration-preparation comparator (1.6100% m/m): RMS deviation** | **Unconstrained composition estimate at Rpw = 5.06% (1.569916% m/m): mean signed deviation** | **Unconstrained composition estimate at Rpw = 5.06% (1.569916% m/m): RMS deviation** | **Forced-origin composition estimate at Rpw = 5.06% (1.471127% m/m): mean signed deviation** | **Forced-origin composition estimate at Rpw = 5.06% (1.471127% m/m): RMS deviation** |
| --- | --- | --- | --- | --- | --- | --- |
| Full MIR | −0.0336 | 0.0520 | +0.0065 | 0.0402 | +0.1053 | 0.1125 |
| PhosCov | −0.0554 | 0.0591 | −0.0153 | 0.0256 | +0.0834 | 0.0859 |
| Amide I | −0.0548 | 0.0994 | −0.0147 | 0.0842 | +0.0841 | 0.1181 |
| Amide II | +0.0058 | 0.0853 | +0.0459 | 0.0967 | +0.1447 | 0.1679 |
| Amide III | −0.0514 | 0.0821 | −0.0113 | 0.0650 | +0.0875 | 0.1084 |
| Amide ALL | −0.0459 | 0.0719 | −0.0058 | 0.0557 | +0.0930 | 0.1082 |
| Amide I_II_III | −0.0316 | 0.0758 | +0.0085 | 0.0694 | +0.1073 | 0.1275 |

Note. All entries are derived from the seven observations in Supplementary Table S1. The 1.6100% m/m value is the separate S2W calibration-preparation Kjeldahl comparator. The two composition-based values are sensitivity scenarios, not assays of the warming liquid. Mean signed deviation is the arithmetic mean of prediction minus comparator; root-mean-square deviation is the square root of the mean squared comparator deviations.

Supplementary Table S7A. Model configuration and leave-one-formulation-out validation summary for the seven spectral-window PLS calibration models.

| **Model** | **Calibration dataset** | **Exact descending calibration axis (cm⁻¹)** | **n variables** | **Algorithm** | **LVs assessed** | **Retained LV** | **LV at min RMSECV** | **Minimum RMSECV** | **RMSEC at retained LV** | **RMSECV at retained LV** | **R²CV at retained LV** |
| --- | --- | --- | --- | --- | --- | --- | --- | --- | --- | --- | --- |
| Full MIR | DataComb_FullMIRSpectra_DTS | 3993.3552-599.5166 | 1654 | SIMPLS | 1-3 | 1 | 1 | 0.091918 | 0.063732 | 0.091918 | 0.996247 |
| PhosCov | DataComb_PhosCov_DTS | 1100.4825-1061.4728 | 20 | SIMPLS | 1-3 | 1 | 2 | 0.041052 | 0.047774 | 0.069934 | 0.997827 |
| Amide I | DataComb_Amide_I_DTS | 1699.9990-1601.4484 | 49 | SIMPLS | 1-3 | 1 | 1 | 0.235441 | 0.136283 | 0.235441 | 0.975375 |
| Amide II | DataComb_Amide_II_DTS | 1601.4484-1500.8446 | 50 | SIMPLS | 1-3 | 1 | 3 | 0.163918 | 0.112603 | 0.187141 | 0.984442 |
| Amide III | DataComb_Amide_III_DTS | 1350.9654-1199.0331 | 75 | SIMPLS | 1-3 | 1 | 1 | 0.102448 | 0.071233 | 0.102448 | 0.995338 |
| Amide I_II_III | DataComb_Amide_I_II_III_DTS | 1699.9990-1201.0863 | 244 | SIMPLS | 1-3 | 1 | 1 | 0.143455 | 0.089163 | 0.143455 | 0.990858 |
| Amide ALL | DataComb_Amide_ALL_PhosCas_DTS | 3299.3943-3278.8629; 3104.3461-3085.8678; 1699.9990-1500.8446; 1350.9654-1201.0863; 1100.4825-1061.4728; 769.9271-630.3137 | 282 | SIMPLS | 1-3 | 1 | 1 | 0.096031 | 0.066501 | 0.096031 | 0.995903 |

Note. The calibration unit is the independently prepared formulation (n = 8), with one retained spectrum per formulation. SIMPLS, X mean centring and single-response Y autoscaling were fitted within each training fold. One latent variable was fixed a priori to limit complexity and permit like-for-like window comparison; LV1-LV3 are sensitivity diagnostics. Single-response autoscaling is multiplication by one constant; after back-transformation, predictions and error metrics remain on the original scale.

Supplementary Table S7B. Leave-one-formulation-out predictions for eight calibration formulations and seven MIR spectral models.

| **Model** | **Omitted formulation** | **Calibration row** | **Measured Kjeldahl protein (% m/m)** | **LOFO prediction (% m/m)** | **Residual: predicted - measured (percentage points)** | **Squared residual (percentage points²)** | **LV** |
| --- | --- | --- | --- | --- | --- | --- | --- |
| Full MIR | S1W | 1 | 3.0000 | 2.998634 | −0.001366 | 0.00000187 | 1 |
| Full MIR | S2W | 2 | 1.6100 | 1.541904 | −0.068096 | 0.00463702 | 1 |
| Full MIR | S3W | 3 | 4.3000 | 4.278127 | −0.021873 | 0.00047843 | 1 |
| Full MIR | S4W | 4 | 0.3400 | 0.451116 | +0.111116 | 0.01234666 | 1 |
| Full MIR | S5W | 5 | 0.9800 | 1.096054 | +0.116054 | 0.01346851 | 1 |
| Full MIR | S6W | 6 | 2.1800 | 2.089069 | −0.090931 | 0.00826849 | 1 |
| Full MIR | S7W | 7 | 2.7200 | 2.632497 | −0.087503 | 0.00765682 | 1 |
| Full MIR | S9W | 8 | 5.0700 | 5.213992 | +0.143992 | 0.02073382 | 1 |
| PhosCov | S1W | 1 | 3.0000 | 3.000322 | +0.000322 | 0.00000010 | 1 |
| PhosCov | S2W | 2 | 1.6100 | 1.550399 | −0.059601 | 0.00355231 | 1 |
| PhosCov | S3W | 3 | 4.3000 | 4.296430 | −0.003570 | 0.00001274 | 1 |
| PhosCov | S4W | 4 | 0.3400 | 0.445027 | +0.105027 | 0.01103072 | 1 |
| PhosCov | S5W | 5 | 0.9800 | 1.038345 | +0.058345 | 0.00340414 | 1 |
| PhosCov | S6W | 6 | 2.1800 | 2.111315 | −0.068685 | 0.00471762 | 1 |
| PhosCov | S7W | 7 | 2.7200 | 2.657679 | −0.062321 | 0.00388385 | 1 |
| PhosCov | S9W | 8 | 5.0700 | 5.181915 | +0.111915 | 0.01252499 | 1 |
| Amide I | S1W | 1 | 3.0000 | 2.970576 | −0.029424 | 0.00086579 | 1 |
| Amide I | S2W | 2 | 1.6100 | 1.517683 | −0.092317 | 0.00852250 | 1 |
| Amide I | S3W | 3 | 4.3000 | 3.955092 | −0.344908 | 0.11896156 | 1 |
| Amide I | S4W | 4 | 0.3400 | 0.481200 | +0.141200 | 0.01993739 | 1 |
| Amide I | S5W | 5 | 0.9800 | 1.079902 | +0.099902 | 0.00998043 | 1 |
| Amide I | S6W | 6 | 2.1800 | 2.231270 | +0.051270 | 0.00262865 | 1 |
| Amide I | S7W | 7 | 2.7200 | 2.626209 | −0.093791 | 0.00879670 | 1 |
| Amide I | S9W | 8 | 5.0700 | 5.593228 | +0.523228 | 0.27376762 | 1 |
| Amide II | S1W | 1 | 3.0000 | 2.997369 | −0.002631 | 0.00000692 | 1 |
| Amide II | S2W | 2 | 1.6100 | 1.524641 | −0.085359 | 0.00728622 | 1 |
| Amide II | S3W | 3 | 4.3000 | 4.089712 | −0.210288 | 0.04422084 | 1 |
| Amide II | S4W | 4 | 0.3400 | 0.458478 | +0.118478 | 0.01403706 | 1 |
| Amide II | S5W | 5 | 0.9800 | 1.156154 | +0.176154 | 0.03103012 | 1 |
| Amide II | S6W | 6 | 2.1800 | 2.103918 | −0.076082 | 0.00578849 | 1 |
| Amide II | S7W | 7 | 2.7200 | 2.601920 | −0.118080 | 0.01394285 | 1 |
| Amide II | S9W | 8 | 5.0700 | 5.474797 | +0.404797 | 0.16386063 | 1 |

**Supplementary Table S7B continued. Leave-one-formulation-out predictions for the remaining MIR spectral models.**

| **Model** | **Omitted formulation** | **Calibration row** | **Measured Kjeldahl protein (% m/m)** | **LOFO prediction (% m/m)** | **Residual: predicted - measured (percentage points)** | **Squared residual (percentage points²)** | **LV** |
| --- | --- | --- | --- | --- | --- | --- | --- |
| Amide III | S1W | 1 | 3.0000 | 3.027951 | +0.027951 | 0.00078127 | 1 |
| Amide III | S2W | 2 | 1.6100 | 1.533216 | −0.076784 | 0.00589575 | 1 |
| Amide III | S3W | 3 | 4.3000 | 4.254696 | −0.045304 | 0.00205249 | 1 |
| Amide III | S4W | 4 | 0.3400 | 0.435710 | +0.095710 | 0.00916046 | 1 |
| Amide III | S5W | 5 | 0.9800 | 1.108866 | +0.128866 | 0.01660648 | 1 |
| Amide III | S6W | 6 | 2.1800 | 2.086029 | −0.093971 | 0.00883047 | 1 |
| Amide III | S7W | 7 | 2.7200 | 2.617095 | −0.102905 | 0.01058950 | 1 |
| Amide III | S9W | 8 | 5.0700 | 5.243343 | +0.173343 | 0.03004796 | 1 |
| Amide I_II_III | S1W | 1 | 3.0000 | 3.000234 | +0.000234 | 0.00000005 | 1 |
| Amide I_II_III | S2W | 2 | 1.6100 | 1.518953 | −0.091047 | 0.00828960 | 1 |
| Amide I_II_III | S3W | 3 | 4.3000 | 4.158293 | −0.141707 | 0.02008079 | 1 |
| Amide I_II_III | S4W | 4 | 0.3400 | 0.451264 | +0.111264 | 0.01237977 | 1 |
| Amide I_II_III | S5W | 5 | 0.9800 | 1.122659 | +0.142659 | 0.02035165 | 1 |
| Amide I_II_III | S6W | 6 | 2.1800 | 2.115146 | −0.064854 | 0.00420609 | 1 |
| Amide I_II_III | S7W | 7 | 2.7200 | 2.618446 | −0.101554 | 0.01031315 | 1 |
| Amide I_II_III | S9W | 8 | 5.0700 | 5.368350 | +0.298350 | 0.08901287 | 1 |
| Amide ALL | S1W | 1 | 3.0000 | 3.017665 | +0.017665 | 0.00031204 | 1 |
| Amide ALL | S2W | 2 | 1.6100 | 1.548992 | −0.061008 | 0.00372193 | 1 |
| Amide ALL | S3W | 3 | 4.3000 | 4.253790 | −0.046210 | 0.00213537 | 1 |
| Amide ALL | S4W | 4 | 0.3400 | 0.428374 | +0.088374 | 0.00780999 | 1 |
| Amide ALL | S5W | 5 | 0.9800 | 1.104338 | +0.124338 | 0.01546000 | 1 |
| Amide ALL | S6W | 6 | 2.1800 | 2.094367 | −0.085633 | 0.00733300 | 1 |
| Amide ALL | S7W | 7 | 2.7200 | 2.617829 | −0.102171 | 0.01043901 | 1 |
| Amide ALL | S9W | 8 | 5.0700 | 5.232987 | +0.162987 | 0.02656484 | 1 |

Supplementary Table S7C. Individual and cumulative X and Y variance explained by LV1-LV3 in the all-eight-formulation diagnostic fits.

| **Model** | **LV** | **X variance individual (%)** | **X variance cumulative (%)** | **Y variance individual (%)** | **Y variance cumulative (%)** | **Active retained LV** |
| --- | --- | --- | --- | --- | --- | --- |
| Full MIR | 1 | 98.6797 | 98.6797 | 99.8196 | 99.8196 | Yes |
| Full MIR | 2 | 0.4052 | 99.0848 | 0.1195 | 99.9390 | No |
| Full MIR | 3 | 0.3584 | 99.4432 | 0.0485 | 99.9875 | No |
| PhosCov | 1 | 99.9953 | 99.9953 | 99.8986 | 99.8986 | Yes |
| PhosCov | 2 | 0.0035 | 99.9988 | 0.0694 | 99.9680 | No |
| PhosCov | 3 | 0.0003 | 99.9991 | 0.0238 | 99.9918 | No |
| Amide I | 1 | 99.8821 | 99.8821 | 99.1749 | 99.1749 | Yes |
| Amide I | 2 | 0.0187 | 99.9008 | 0.6301 | 99.8051 | No |
| Amide I | 3 | 0.0154 | 99.9162 | 0.1400 | 99.9451 | No |
| Amide II | 1 | 99.9380 | 99.9380 | 99.4367 | 99.4367 | Yes |
| Amide II | 2 | 0.0188 | 99.9568 | 0.5082 | 99.9450 | No |
| Amide II | 3 | 0.0100 | 99.9668 | 0.0516 | 99.9966 | No |
| Amide III | 1 | 99.9484 | 99.9484 | 99.7746 | 99.7746 | Yes |
| Amide III | 2 | 0.0103 | 99.9587 | 0.1953 | 99.9699 | No |
| Amide III | 3 | 0.0071 | 99.9658 | 0.0273 | 99.9972 | No |
| Amide I_II_III | 1 | 99.8439 | 99.8439 | 99.6468 | 99.6468 | Yes |
| Amide I_II_III | 2 | 0.0858 | 99.9297 | 0.1834 | 99.8302 | No |
| Amide I_II_III | 3 | 0.0275 | 99.9571 | 0.1497 | 99.9799 | No |
| Amide ALL | 1 | 99.2466 | 99.2466 | 99.8035 | 99.8035 | Yes |
| Amide ALL | 2 | 0.3166 | 99.5632 | 0.1071 | 99.9106 | No |
| Amide ALL | 3 | 0.1738 | 99.7370 | 0.0719 | 99.9825 | No |

Note. Percentages are PLS_Toolbox detail.ssq values from all-eight-formulation calibration fits. One latent variable was retained a priori for all active models. LV2 and LV3 are sensitivity diagnostics only; leave-one-formulation-out RMSECV is reported separately. The 21 rows are model diagnostics, not additional experimental observations.

Supplementary Table S8. Row-level PhosCov leave-one-temperature-out internal correction calculations.

| **Temperature (°C)** | **Original indication (% m/m)** | **Formulation-associated comparator (% m/m)** | **Original comparator deviation (percentage points)** | **Linear LOTO-predicted comparator deviation (percentage points)** | **Linear corrected indication (% m/m)** | **Linear corrected comparator deviation (percentage points)** | **Quadratic LOTO-predicted comparator deviation (percentage points)** | **Quadratic corrected indication (% m/m)** | **Quadratic corrected comparator deviation (percentage points)** |
| --- | --- | --- | --- | --- | --- | --- | --- | --- | --- |
| 2.0 | 1.553535 | 1.610000 | -0.056465 | -0.027582 | 1.581117 | -0.028883 | -0.071064 | 1.624599 | 0.014599 |
| 5.0 | 1.569781 | 1.610000 | -0.040219 | -0.048038 | 1.617819 | 0.007819 | -0.048038 | 1.617819 | 0.007819 |
| 8.0 | 1.551780 | 1.610000 | -0.058220 | -0.048963 | 1.600742 | -0.009258 | -0.031657 | 1.583437 | -0.026563 |
| 11.0 | 1.571930 | 1.610000 | -0.038070 | -0.058321 | 1.630250 | 0.020250 | -0.041368 | 1.613298 | 0.003298 |
| 14.0 | 1.560390 | 1.610000 | -0.049610 | -0.062550 | 1.622940 | 0.012940 | -0.048574 | 1.608964 | -0.001036 |
| 17.0 | 1.566949 | 1.610000 | -0.043051 | -0.073852 | 1.640801 | 0.030801 | -0.073852 | 1.640801 | 0.030801 |
| 20.0 | 1.507642 | 1.610000 | -0.102358 | -0.041702 | 1.549343 | -0.060657 | -0.045466 | 1.553108 | -0.056892 |

Note. LOTO = leave-one-temperature-out. Corrected indications were generated internally from the same seven-observation trajectory using the formulation-associated comparator. They are illustrative calculations, not independent validation results or a transferable correction model.

**Supplementary Figures**

Supplementary Figure S1. Distribution of the seven ordered warming-sequence predictions for each MIR model. Horizontal segments denote model means; the dashed line denotes the 1.6100% m/m formulation-associated comparator from the separate S2W calibration preparation. No inferential comparison is implied. The plotted % unit denotes % m/m.

Supplementary Figure S2. Full-range overlay of low-solids ATR-MIR spectra acquired sequentially at 2, 5, 8, 11, 14, 17 and 20 °C during one passive-warming trajectory. The spectra are plotted on a common absorbance scale to show within-trajectory temperature-associated changes.

Supplementary Figure S3A. Leave-one-formulation-out RMSECV as a function of latent-variable count. One latent variable was fixed a priori for the reported models; LV2 and LV3 are sensitivity diagnostics only.

Supplementary Figure S3B. Measured Kjeldahl protein versus leave-one-formulation-out prediction for the retained one-latent-variable models. Each point represents one withheld calibration formulation; the dashed line is the line of identity. The plotted % unit denotes % m/m.

Supplementary Figure S3C. Leave-one-formulation-out residuals for the retained one-latent-variable models. Each point represents one withheld calibration formulation; residual equals predicted minus measured protein.

Supplementary Table S0B. Calibration formulations and independently determined Kjeldahl protein values.

| **Formulation** | **Skim-milk powder (g)** | **Distilled water (g)** | **Powder-to-water ratio (%)** | **Kjeldahl protein (% m/m)** |
| --- | --- | --- | --- | --- |
| S4W | 2.51 | 250.50 | 1.00 | 0.34 |
| S5W | 7.64 | 250.44 | 3.05 | 0.98 |
| S2W | 12.66 | 250.20 | 5.06 | 1.61 |
| S6W | 17.61 | 250.10 | 7.04 | 2.18 |
| S7W | 22.40 | 250.29 | 8.95 | 2.72 |
| S1W | 25.14 | 250.75 | 10.03 | 3.00 |
| S3W | 37.44 | 250.72 | 14.93 | 4.30 |
| S9W | 45.17 | 250.10 | 18.06 | 5.07 |

Note. Protein was reported on a % m/m basis using laboratory method PROT010201 and the total-nitrogen-to-protein factor 6.38. One retained spectrum per independently prepared formulation was used in the calibration data matrices; the laboratory aliquot came from the same prepared formulation.

Supplementary Figure S4. Full-range absorbance spectra retained for the eight independently prepared calibration formulations. Legend values are the corresponding independently determined Kjeldahl protein concentrations.

Source: 01_Calibration/Calibration_Spectral_CSV/CalibrationSpectra_01_FullMIR_DataComb_FullMIRSpectra_DTS_LONG.csv and 01_Calibration/Calibration_Formulations_and_Kjeldahl.csv.
